# Early treatment of acute hepatitis C infection is cost-effective in HIV-infected men-who-have-sex-with-men

**DOI:** 10.1371/journal.pone.0210179

**Published:** 2019-01-10

**Authors:** Stephanie Popping, Sebastiaan J. Hullegie, Anne Boerekamps, Bart J. A. Rijnders, Robert J. de Knegt, Jürgen K. Rockstroh, Annelies Verbon, Charles A. B. Boucher, Brooke E. Nichols, David A. M. C. van de Vijver

**Affiliations:** 1 Department of Viroscience, Erasmus MC, Rotterdam, The Netherlands; 2 Department of Internal Medicine and Infectious Diseases, Erasmus MC, Rotterdam, The Netherlands; 3 Department of Gastroenterology and Hepatology, Erasmus MC, Rotterdam, The Netherlands; 4 Department of Internal Medicine, Bonn University Hospital, Bonn, Germany; 5 Department of Global Health, Boston University, Boston, United States; Centers for Disease Control and Prevention, UNITED STATES

## Abstract

**Background:**

Treatment of hepatitis C virus infections (HCV) with direct acting antivirals (DAA) can prevent new infections since cured individuals cannot transmit HCV. However, as DAAs are expensive, many countries defer treatment to advances stages of fibrosis, which results in ongoing transmission. We assessed the epidemiological impact and cost-effectiveness of treatment initiation in different stages of infection in the Netherlands where the epidemic is mainly concentrated among HIV-infected MSMs.

**Methods:**

We calibrated a deterministic mathematical model to the Dutch HCV epidemic among HIV-infected MSM to compare three different DAA treatment scenarios: 1) immediate treatment, 2) treatment delayed to chronic infection allowing spontaneous clearance to occur, 3) treatment delayed until F2 fibrosis stage. All scenarios are simulated from 2015 onwards. Total costs, quality adjusted life years (QALY), incremental cost-effectiveness ratios (ICERs), and epidemiological impact were calculated from a providers perspective over a lifetime horizon. We used a DAA price of €35,000 and 3% discounting rates for cost and QALYs.

**Results:**

Immediate DAA treatment lowers the incidence from 1.2/100 person-years to 0.2/100 person-years (interquartile range 0.1–0.2) and the prevalence from 5.0/100 person-years to 0.5/100 person-years (0.4–0.6) after 20 years. Delayed treatment awaiting spontaneous clearance will result in a similar reduction. However, further delayed treatment to F2 will increases the incidence and prevalence. Earlier treatment will cost society €68.3 and €75.1 million over a lifetime for immediate and awaiting until the chronic stage, respectively. The cost will increase if treatment is further delayed until F2 to €98.4 million. Immediate treatment will prevent 7070 new infections and gains 3419 (3019–3854) QALYs compared to F2 treatment resulting in a cost saving ICER. Treatment in the chronic stage is however dominated.

**Conclusions:**

Early DAA treatment for HIV-infected MSM is an excellent and sustainable tool to meet the WHO goal of eliminating HCV in 2030.

## Introduction

Treatment of hepatitis C virus (HCV) infections has dramatically improved since the advent of well-tolerated direct acting antivirals (DAAs). DAA treatment results in a 90–95% sustained virological response (SVR), which is associated with strongly reduced morbidity and cure[1, 2]. Importantly, as individuals that are cured cannot transmit HCV to others, DAAs can be used as prevention strategy. Apart from modeling studies, this was shown in a recent study in the Netherlands where new HCV infections were reduced by 70% after widespread use of DAAs [3, 4]. The World Health Organization (WHO) shares the optimism that DAAs can prevent new infections and declared an ambitious target of ending HCV as a public health treat in 2030[5].

A key challenge in prevention of HCV is the timing of start of DAA treatment. As DAAs are expensive, many countries defer treatment to advances stages of fibrosis, which can result in continued transmission of the virus[6]. In countries that reimburse expensive DAAs, patients usually start treatment several months after the presumed date of infection to allow spontaneous clearance (15–20% of patients) [7, 8]. Importantly, high risk individuals can continue HCV transmission during that time frame.

In this study we assessed the epidemiological impact and cost-effectiveness of start of treatment in different stages of infection. For this purpose, we used the Netherlands, where HIV-infected MSMs account for 94% of the new HCV infections[9]. MSM are at high risk of acquiring HCV due to high risk-sexual behavior, including an excessive number of partners combined with drug use[10]. In the Netherlands, contrary to many other countries, no new HCV infections are reported among injecting drug users (IDU)[11, 12]. A key advantage of the Netherlands is that DAAs are reimbursed for all HCV stages since 2016. However, before 2016, use of DAAs was restricted to METAVIR F2 stage. The epidemiological impact of DAAs has been reported for the scenario where DAAs were restricted to advanced stages of fibrosis (before 2016) and after DAAs were used irrespective of the stage of fibrosis (after 2016)[3, 13]. Therefore, we could calibrate our model to the epidemiological impact of unrestricted DAA treatment after a period of restricted DAAs by assuming that the incidence of HCV would remain comparable to the epidemic before 2016 and we could calibrate the model to the scenario of unrestricted DAAs by including the epidemiological impact after 2016.

## Methods

### Study design and population

The HIV epidemic in the Netherlands is concentrated among MSM, with nearly 94% of infected patients reporting MSM as the mode of transmission, making it very similar to the HIV epidemic in other high-income countries[14, 15]. This young epidemic is characterized with incidence rates of 1/100 persons-years[16, 17]. In addition, HCV reinfections are a major concern in this population, with incidence rates of 7.3/100 person-years after cure[18]. The epidemic is well described through a national HIV database (ATHENA cohort), which contains anonymized demographic and clinical data of >98% of patients in HIV-care in the 27 treatment centers in the Netherlands[19]. We developed a deterministic mathematical model to represent the HCV/HIV epidemic among MSM in the Netherlands.

### Model parametrisation and calibration

We calibrated our model to the Dutch HIV epidemic including data on HCV from the Dutch Acute HCV in HIV study (DAHHS) [13, 19–22]. Our calibration is based on the estimated Dutch MSM population size, the percentage of individuals co-infected with HCV, a stable HCV incidence rate of 1.2 per 100 person-years, and a reinfection rate of 15 per 100 PY (range 8 to 26.5 per 100 PY)[13, 21, 23–25] (Table 1, S1 Fig. S1 Text). We accounted for the population effect of widespread DAA use by validating our model’s projected incidence in 2016 with published Dutch HCV incidence data of 2016 (0.4–1.0/100 PY) [3, 26]. With Monte Carlo filtering techniques a total of 132 out of 100,000 simulations remained that matched the Dutch HCV epidemic among HIV-infected MSM[27–29] (S1 Table).

**Table 1:** Model parameters and ranges used in hepatitis C (HCV) transmission model.

Model Parameters of HCV transmission model among Dutch MSM	Range/number (median) ƚ = calibrated
Annual HIV diagnoses among MSM per time period	
2002–2014	720–740[20]
2015	620[19]
2016	580
Susceptible HIV infected MSMs in 2002	3800
Patients with HCV in 2002	2–10%[25] ƚ
Mortality rate HIV patients ≥350 CD4 count	1/45[31] *
Transmissibility of HCV	0.01–0.05 ƚ
Diagnosed percentage per HCV testing moment	70–100%[32, 33]
Clearance rate	15–25%[7, 8, 34]
Time to clearance	40–170 days[41]
Reinfection rate	8–26.5%, per year[35, 36]
Time from transmission until treatment (acute HCV)	16.5–25 weeks[37]
Time from transmission until treatment (F0 chronic)	20.4–54.2 weeks [37] ƚ ¥
Patients in stage F3, F4 in 2002	10–30%
HCC rate	2–5% [38, 39]
Treatment parameters	**Range/number**
SVR, DAA F0-F3	89–100%[40, 41]
Treatment duration F0-F3	12 weeks[42]
SVR, DAA cirrhosis	80–95% [43]
Treatment duration F4 compensated and decompensated	16 weeks[44]
Retreatment duration F0-F3	12 weeks[42]
Retreatment duration F4 compensated and decompensated	16 weeks[44]
Quality of Life	**Utility score**
HIV mono-infection	0.94[45]
Acute HCV infection	0.84[37]§
HCV F0-F3 stage	0.84[46]§
Compensated cirrhosis	0.38–0.67[47]
Decompensated cirrhosis	0.38[47]
DAA based therapy	0.84[37]§
Costs	**Price in €**
Doctors visit	€136[48]
HCV RNA	€105-€225^¶^
HCV genotype	€130-€252^¶^
Ultrasound of the liver	€90-€226^¶^
Biochemistry and liver function tests	€38-€46^¶^
F3 additional costs per year **	€807.88^¶^
F4 additional costs per year **	€807.88^¶^
DAA regimen 12 weeks	€35,000^¶^

Abbreviations: HCV: hepatitis C virus,MSM: men having sex with men,SVR: sustained virological response,PEG-IFN: PEGylated interferon,RBV: ribavirin, DAA: direct-acting antiviral.

* Successfully treated patients who achieved viral suppression and attained a CD4^+^ cell count of at least 350 cells/μl within 1 year of starting ART had a normal life expectancy, with a 35-year-old HIV-positive person estimated to live to about 80 years on average.

** Additional cost per year are based on the abdominal echo’s (HCC screening), additional doctor appointments and biochemistry.

¥ Weeks are based on the time that a patient needs to be diagnosed (16.5–25 weeks[37]) with an additional number of weeks that is “waited” until a patient reach possible spontaneous clearance. In the model we “wait” an additional 3–3.5 months for spontaneously clearance (+/- 90 days).

§ The model considers the HCV/HIV co-infection utility score to be an interaction between the utility for HIV mono and HCV mono scores. The utility scores are varied in the sensitivity analysis.

^¶^ Dutch data summarized out of different academic hospitals in the Netherlands.

Our model stratifies disease progression into individuals that spontaneously clear the virus (15–20% of cases[7]), three stages of progressive fibrosis (METAVIR stages F0-F3), and two stages of cirrhosis (stage F4 sub-divided in compensated- and decompensated cirrhosis). From stage F3, F4 compensated and F4 decompensated cirrhosis patients can develop a hepatocellular carcinoma (HCC) with a rate of 2–5%.

The rate by which HCV/HIV co-infected individuals progress from a particular stage of fibrosis to a more advanced stage of fibrosis is approximately 10% per year (this rate of progression results in a probability of having cirrhosis–stage F4- of 20.8% to 48.5% after 20 to 30 years, respectively)[30] (S2 Table). Due to a shortage of donors, liver transplantation has not been performed in HIV/HCV co-infected individuals in the Netherlands and is, therefore not considered in the model. We assumed that during HCV treatment individuals are virological suppressed and do not transmit HCV to others. In our model before 2012, chronically infected patients in F2 through F4 fibrosis stages were treated with pegylated interferon and ribavirin. Between 2012 and 2015, boceprevir or telaprevir in addition to pegylated interferon and ribavirin, was prescribed to chronically infected patients. We assumed that until 2015, between 67% and 75% of patients were treated for 24 weeks with pegylated interferon and ribavirin (other patients declined treatment) as in agreement with the treatment guidelines that were in place. After 2015, pegylated interferon was no longer considered, since DAAs were reimbursed for all stages of HCV infection in the Netherlands.

In our model there are four different risk groups in which individuals have a different number of HIV-infected partners per years [28](S2 Table).

### Different treatment scenarios

All HIV-infected MSM undergo HCV screening, using a biannual ALT and annual antibody test, [49] in which the model assumes that approximate 85% of the HCV infections are diagnosed[32, 33]. After diagnosis, treatment is given according to three treatment scenarios evaluated in the model from 2015 onwards. In the first scenario DAAs are given immediately after diagnosis in the acute stage of HCV (immediate treatment). The model accounts a median time of 18.1 weeks (range 16.5–25) from transmission until treatment initiation of acute HCV[22]. In the second scenario, treatment is delayed until the chronic stage, awaiting spontaneous clearance varying from 40–170 days[41] (chronic treatment). In the third scenario DAAs are delayed until an advanced stage of HCV infection, F2 METAVIR (delayed F2 treatment) (S2 Fig).

In our model all individuals with that do not have cirrhosis receive a 12-week DAA treatment course. SVR rates for treatment ranged between 89–100% with a median of 94% (Table 1). If a SVR is not achieved individuals are re-treated with a 12-week DAA course. During the cirrhotic stage DAA treatment is prolonged until 16 weeks with SVR rates for treatment between 80–95%[43].

### Cost and QALY estimates

The cost-effectiveness analysis was performed from a provider perspective. Each compartment in our deterministic model was assigned a cost and quality adjusted life year (QALY) score (Table 1). Costs for HCV monitoring and treatment were collected among the six Academic Medical Centers in the Netherlands. Our model used a DAA price of €35,000 for a 12-week treatment course, which is varied in the sensitivity analysis. QALY weights were obtained from data of the Dutch HIV/HCV co-infected MSM cohort (DAHHS)[37]. HIV mono-infected MSM are assumed to have a QALY of 0.94[45]. The model considers the HCV/HIV co-infection utility-score to be an interaction between the HIV- mono and HCV-mono infected utility scores. HCV/HIV co-infected MSM are assumed to have a utility score of 0.84 during F0-F3 stage. QALY-scores during DAA treatment remained similar. After resolving the HCV infection, the QALY-score returned to that of an HIV mono-infected (i.e. 0.94 [45]). Both costs and QALY-scores were discounted at 3% per year[50, 51]. For this study, we used a willingness-to-pay threshold of €20,000 per QALY[50, 52].

HIV-infected MSM are co-infected with HCV at a median age of 40 years[37]. In addition, an HIV-infected MSM with CD4 >350 cells/μl has a life expectancy of 80 years[31]. Therefore, we used a 40-year time horizon to calculate the epidemiological impact and economic outcomes[53]. The reported numbers are the median values with the corresponding interquartile range between brackets. Prices are notated in euros (€).

### Sensitivity analysis and uncertainties

We performed a one-way sensitivity analysis of the incremental cost-effectiveness ratios comparing the immediate treatment scenario with the delayed F2 treatment scenario. Several key input variables were varied: cost of DAAs (€5,000 - €50,000), spontaneous clearance rate (5–10% - 15–30%), discounting rates (0–5%), HCV testing intervals (3–12 months), QALY-score during DAA treatment (0.84–0.94), an increase in the number of high-risk MSM that are at risk of acquiring HCV (up to 6500 individuals) since the introduction of HIV pre-exposure prophylaxis (PrEP) and the impact of continuing transmission from undiagnosed HCV infected individuals (up to 100 individuals that remain undiagnosed)[54]. HIV PrEP users should be taken into account since HCV prevalence among HIV-negative PrEP users is increasing, in contrast to a stabilizing prevalence among HIV-negative MSM [26, 55–57]. As data that we could use for calibration of HCV among HIV-uninfected MSM and PrEP users is not fully available we established a sensitivity analysis.

## Results

### Model projections

#### Epidemiological impact of different treatment scenarios

**Fig 1:**
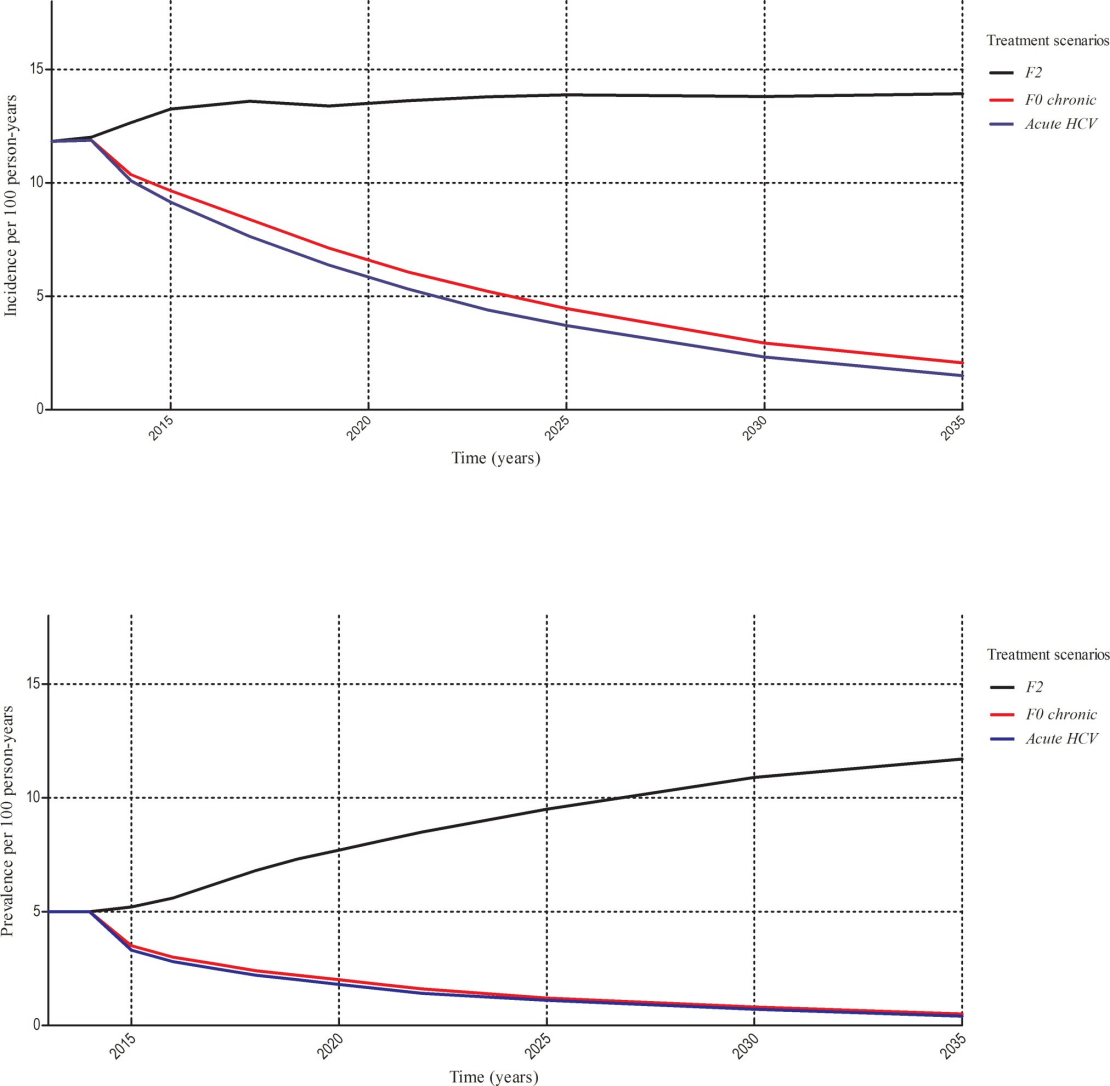
Short term epidemiological hepatitis c virus impact among HIV positive men who have sex with men. In the upper figure, the hepatitis C virus incidence is projected and in the lower figure the hepatitis C virus prevalence. Three different treatment scenarios were simulated over a short term period of 20 years. F2, delaying treatment until a F2 fibrosis stage. F0 chronic, awaiting the time frame of spontaneously clearance. Acute HCV, treatment in the acute stage. Median rates are reported. Abbreviations: HCV = hepatitis C virus.

Before 2015 there was a stabilizing incidence of 1.2/100 person-years and prevalence of 5.0%. After starting our treatment scenarios, the model projected an increasing, but further stabilizing incidence at 1.4/100 person-years (IQR 1.2–1.7) for the delayed F2 treatment scenario after 20 years. The prevalence is projected to increase over time to 9.5% (8.8–10.5) in 2025 and to 11.7% (10.3–13.3) in 2035 (Fig 1A and 1B).

Treatment in the chronic stage of infection, after awaiting clearance, will reduce the incidence by 68% to 0.5/100 person-years (0.4–0.5) in 2025 and by 84% in 2035 to 0.2 /100 person-years (0.2–0.3) compared to delayed F2 treatment. The prevalence will reduce over time by 87% to 1.2% (1.1–1.4) in 2025 and by 95% to 0.5% (0.4–0.7) in 2035. Over the 40 year time horizon, a total of 7070 new infections were prevented in the chronic treatment scenario as to compared to the delayed F2 treatment scenario.

Immediate treatment will further reduce the incidence by 73% to 0.4/100 person-years (0.3–0.4) in 2025 and by 88% to 0.2/100 person-years (0.1–0.2) in 2035 and the prevalence by 89% to 1.0% (0.9–1.1) in 2025 and by 96% to 0.4% (0.3–0.5) in 2035. A total number of 7457 new infections were prevented by immediate treatment compared to delayed F2 treatment over 40 years.

### Impact of different treatment scenarios on Hepatocellular carcinoma

Our model projected an increasing HCC incidence rate for delayed F2 treatment up to 2032 before it slowly stabilizes and starts to decrease. This increase is also attributed to the removal of pegylated interferon as treatment for acute HCV infections. More individuals will therefore enter an F3 stage and are at risk for HCC. Delayed F2 treatment will result in an HCC incidence of 0.42 per 1000 person-years (IQR 0.28–0.59). Immediate treatment and chronic treatment will dramatically reduce the incidence rates to 0.01 per 1000 person-years (0.00–0.02) and 0.01 per 1000 person-years (0.01–0.03) after 40 years, respectively. (S3 Fig).

### Cost-effectiveness

Our model projected that the HCV epidemic among Dutch HIV co-infected MSMs would cost €98.4 million (IQR €87.9–112.9) with delayed F2 treatment over a lifetime (Table 2).

**Table 2:** Results of the main cost-effectiveness analysis of three different DAA treatment scenarios.

Scenario*	HCV infections averted at 40yr	HCV Prevalence reduction at 20yr	Total costs, Euro’s € (millions)	QALY x 1000	Incremental costs Euro’s € (millions)	Incremental QALYs	ICER
F2	-	-	€ 98.4	331.3	-	-	-
F0 acute	*7457*	*97%*	€ 68.3	334.7	-€ 30.0	3425	cost saving
F0 chronic	*7070*	*96%*	€ 75.1	334.6	€ 6.9	-47	dominated

The reported numbers are median values with the corresponding interquartile ranges between brackets. Abbreviations: HCV: hepatitis C, QALYs: Quality Adjusted Life Years, ICER: incremental cost-effectiveness ratio.

* Scenario F2; DAA treatment is delayed until the F2 stage. Scenario F0 acute; DAA treatment is given in the acute HCV stage. F0 chronic: DAA treatment is delayed until the chronic stage of HCV infection.

However, immediate treatment and treatment according to the chronic scenario would cost far less, €68.3 million (62.9–75.1) and €75.2 million (69.3–84.3) over 40 years, respectively. The projected cost reduction is mainly attributed to the infections prevented by timely initiation of DAA treatment. There were 3,419 QALYs gained (3,019–3,854) in the immediate treatment scenario compared to delayed F2 treatment. This, combined with the lower cost of immediate treatment over the 40 year time horizon, resulted in the immediate treatment scenario being cost-saving (Table 2). The chronic treatment scenario is, however, dominated by immediate treatment, given that chronic treatment was more costly and resulted in fewer QALYs gained than immediate treatment. In addition, awaiting spontaneous clearance and therefore delaying treatment is associated with increased costs €6.9 million and a decrease of 47 (34–71) QALYs as compared to immediate treatment.

### Sensitivity analysis

**Fig 2:**
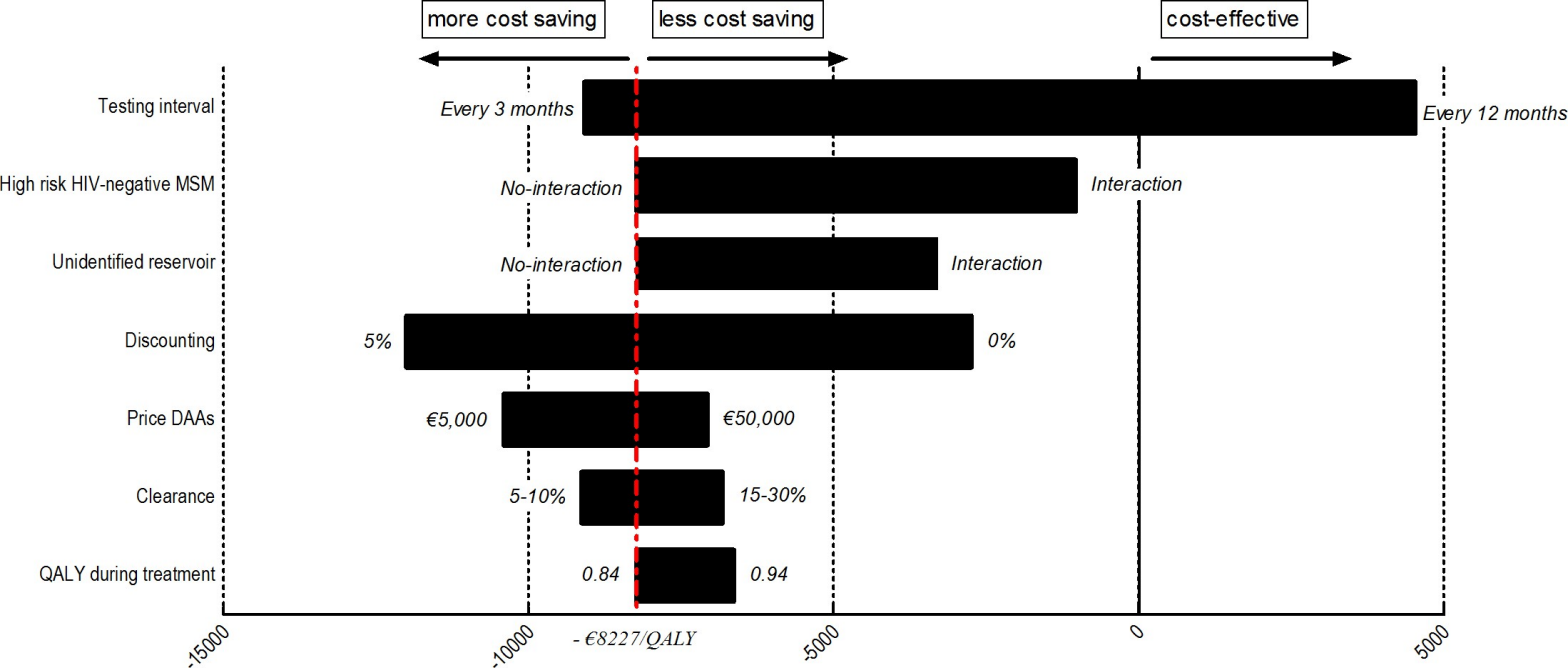
One-way sensitivity analysis of the incremental cost-effectiveness ratio(ICERs) (€/QALY) of direct acting antiviral treatment (DAA). **–** DAA Treatment in the acute stage of infection is compared to delayed F2 DAA treatment with varying different key parameters. The bars show the range in ICER if these key variables are varied. All ICERs are stated in euros. Abbreviations: DAA: direct-acting antivirals, ICER: incremental cost-effectiveness ratio, MSM men-who-have-sex-with-men.

We conducted a one-way sensitivity analysis of the incremental cost-effectiveness ratio (ICERs) of immediate treatment compared to the delayed F2 treatment scenario (Fig 2). The ICER most strongly depends on the testing intervals, and immediate treatment is more cost saving when the testing interval is three-monthly, and cost-effective at €6,348 per QALY gained for annual testing.

Although our sensitivity analysis showed that the precise epidemiological impact of the DAAs on the HCV incidence changes, in both situations immediate start of DAA treatment as compared to delaying to F2 stage remained cost-saving. Hence it is of utmost importance that all high risk MSM, regardless of HIV status, are regularly screened for HCV to maintain the treatment as prevention effect for DAAs. While the DAA price influences the ICER, immediate treatment remains cost-saving.

## Discussion

We used a deterministic mathematical model to compare the economical and epidemiological impact of three different DAA treatment scenarios among HIV-infected MSM in the Netherlands. The key finding of our study is that treatment of acute HCV infections (immediate treatment scenario) is a cost-saving intervention, since immediate treatment will save money and increases health benefits in the long term. Moreover, it will reduce HCV incidence among HIV-infected MSM, despite the high reinfection rates. This strongly indicates that DAAs treatment for acute HCV is a suitable and financially sustainable tool to reach viral hepatitis elimination goals as defined by the WHO (i.e. 90% reduction in new chronic infections and 65% reduction in mortality).

Our study showed that the size of the future HCV epidemic is highly influenced by treatment initiation time. A decrease of incidence and prevalence when treating individuals in earlier stages of the HCV disease is predicted. On the contrary, an increase in incidence, prevalence, and a higher number of HCCs is predicted when further delaying treatment. Therefore, treatment should be administrated in a timely manner to avoid further transmission and to reduce future health care related costs. In addition, awaiting clearance before initiating treatment is less beneficial and not suitable for the HIV co-infected MSM population as compared to immediate treatment upon diagnosis.

Our findings are important for treatment and for public health as they indicate an economic advantage of DAA treatment in the early stages of infection as compared to deferring treatment. In many countries the extraordinarily high cost of DAAs resulted in restrictive reimbursement policies[58]. Restrictions can be based on fibrosis, co-infection and substance abuse[42, 59, 60]. Still, countries continue to delay DAA treatment until F2 or even F3 stages[60, 61]. Our model concludes that limited access and delaying treatment will only increase incidence, prevalence, and related costs.

Several cost-effectiveness studies on the impact of DAAs on HCV were performed among people who inject drugs (PWID). These studies found that DAAs are cost-effective among PWIDs[62–64] However, the results of these studies cannot be compared to our model as PWID are not comparable with HIV-infected MSM. HIV-infected MSM are unlike PWIDs, as they are often well-defined and in regular HIV-care. In addition, risk behavior and reinfection rates differ[65].

Our findings are in agreement with two other modelling studies, one from the United Kingdom and the other from Switzerland, that predicted the epidemiological impact of DAAs on the HCV epidemic among HIV-infected MSM [4, 66]. However, our study measured not only the epidemiological impact, but also the cost-effectiveness. The WHO recommends to conduct cost-effectiveness studies, as one of the pillars in their elimination goals, in order to aim for long-term program sustainability. In addition, due to the new Dutch Acute HCV in HIV incidence data, we were able to measure the population-level effect of the DAAs after an unrestricted roll-out[3].

A key strength of our model is that we are, to our knowledge, the first cost-effectiveness study that includes the population benefits of DAAs started in different stages of fibrosis obtained from a real-world setting[3]. Another strength is that our model is based on data of the well monitored HIV epidemic in the Netherlands[14]. As a consequence, our mathematical model is calibrated to complete and accurate data on the annual number of (newly) diagnosed HIV-infected MSM and data on incident HCV infections among people living with HIV in the Netherlands. Combined, these two strengths allowed us to make accurate predictions of the effect of unrestricted access to DAAs and the effect of deferred treatment, on the HCV epidemic among HIV-infected MSMs on a population level which captures also the “unknown” influence of treatment of other risk groups in the Netherlands interacting with the HIV-infected MSM population.

In our model we did not specify the different DAA regimens and different genotypes. Genotype was known to influence the response to pegylated interferon containing regimens [41, 67, 68]. However, due to the excellent efficacy of the DAAs, regimens have high SVR rates irrespective of the genotype. In our model we used SVR rates of 89–100% which are in agreement with reported ranges for DAA treatment of non-cirrhotic stages of infection (F0-F3). We used a 12 week regimen in our model since most treatment regimens are recommend 12 weeks regardless of genotype or fibrosis stage [42].

The price of DAAs is known to vary between countries and between regimens. We therefore conducted a sensitivity analysis varying the DAA price between €5,000 and €50,000 (Fig 2). A lower DAA price results in a more cost-saving ICER. Our sensitivity analysis also showed that the cost-effectiveness of DAA treatment strongly depends on the HCV testing frequency in routine clinical care. HIV infected MSM are bi-annually screened for HCV. More frequent testing will, however, lead to timely identification of acute HCV infections and more prevented infections[69].

In the Netherlands the epidemic is solely driven by MSM and new infections due to injecting-drug use (IDU) are almost zero [11, 12]. We do realize that there are countries in which IDU remains a problem and that interaction between MSM and IDU may occur. The study of Virologeux et al. assessed the influence of interaction between the IDU population and the HIV-infected MSM population and no difference was found regarding elimination outcome if there would be a limited amount of interaction [70].

In conclusion, our study shows that DAA treatment for acute HCV infected is a cost-saving prevention approach that strongly reduces the HCV epidemic among HIV-infected MSM, despite high reinfection rates. Furthermore, shows our study that although earlier treatment (F0 chronic) is dominated by acute treatment, this is still highly favorable compared to delayed F2 treatment. Concerns about economic sustainability of expensive DAAs should, therefore, not be a reason to restrict DAAs to more advanced stages of fibrosis. Moreover, our study addresses the consequences of delaying treatment in a population with high risk behavior while adequate treatment is available. We concluded that DAAs are an excellent and sustainable tool to meet the WHO elimination goals and that all HIV-infected MSM should have universal accessibility regardless of infectious stage.

## Ethics statement

Data used for calibration of the model from the Dutch HIV cohort and the DAHHS study were fully anonymized. In addition, all patients gave informed consent to have their data used in research projects.

## Supporting information

S1 Table Variables used to calibrate and accept simulations using the Monte Carlo filtering technique. (PDF)

S2 Table Parameters in the model. (PDF)

S1 Fig Calibrated figures. Comparison of the projected number of MSM that are diagnosed with HIV (black bullets and line) and the actual number of MSM diagnosed as reported by the Dutch HIV monitoring foundation. Comparison of the incidence rate of the Dutch population over time and the median simulations of our model. At T = 2014 the first DAAs were introduced in the Netherlands for F2/F3 patients and treatment in clinical trials. In 2015 DAAs became unrestricted available and in 2016 a new incidence data was available.
(PDF)

S2 Fig Simplified diagram capturing the HCV transmission model among HIV infected MSM evaluating different treatment scenarios. Individuals can be treated during an acute HCV infection in the immediate scenario, or treatment is delayed until possible spontaneous clearance, the so called chronic treatment scenario, or individuals are treated according to the delayed F2 treatment scenario. The stage of HCV infection. Individuals progress through the natural course of disease over time. Patients who do not spontaneously clear (Cl) their HCV infection can be put on DAA treatment. Abbreviations: DAA: direct-acting antivirals, F0-F3: fibrosis score METAVIR, HCC: hepatocellular carcinoma.
(PDF)

S3 Fig Cumulative avoided hepatitis C related hepatocellular carcinoma compared to delayed F2 treatment. Simulated Hepatitis C related hepatocellular carcinoma that can be avoided when treatment is administrated timely instead of delayed until F2 stage among HIV positive men-who-have-sex-with-men. F0 chronic, initiating treatment in the chronic phase of infection, waiting for the infection to spontaneously clear. T = 0 start of intervention.
(PDF)

S4 Fig Influence of removal of pegylated-interferon as treatment of acute hepatitis C on the incidence among HIV-positive men-who-have-sex-with-men. In current guidelines pegylated-interferon is no longer recommended in the acute stage of hepatitis C virus (HCV) infection. However, individuals treated and cured with pegylated-interferon during the acute stage of HCV infection, could not further transmit HCV. The removal of pegylated-interferon and delaying DAA treatment until F2, therefore has a negative epidemiological impact. We conducted an uncertainty analysis, in which DAA treatment is delayed until F2 stage and pegylated-interferon is a possible option during the acute stage of HCV. In addition, the incidence decline of immediate and treatment during the F0 chronic stage is projected. Our analysis shows that the removal of pegylated-interferon during the acute stage of HCV infection and delaying treatment until F2 results in an increase of HCV incidence over time. Pegylated-interferon as an optional treatment in the acute stage while awaiting DAA treatment will stabilize the incidence. In contrast, early treatment with DAAs strongly reduces the HCV incidence over time.
(PDF)

S5 Fig One-way sensitivity analysis of higher unidentified HCV reservoir. One-way sensitivity analysis of treatment immediately with DAAs after diagnosis versus delaying DAA treatment until F2 METAVIR stage with a cost-saving ICER of -8227/QALY. In both scenarios we simulate a group of individuals with undiagnosed/untreated HCV, the so called unidentified reservoir. We vary this from 0–100 and 0–2000. In addition, from 2016 onwards 6500 individuals are forced into the high risk groups of our model.
(PDF)

S1 Text Model description. (PDF)

S2 Text Technical model and equations. (PDF)
