# Supplementary material for: Early treatment of acute hepatitis C infection is cost-effective in HIV-infected men-who-have-sex-with-men
Source: PLoS One. 2019 Jan 10;14(1):e0210179. doi: 10.1371/journal.pone.0210179 (PMC6328146; doi:10.1371/journal.pone.0210179)
Supplement: S1 Table — (PDF) [file pone.0210179.s001.pdf]

# S1 Variables used to calibrate and accept simulations using the Monte Carlo filtering technique

| Parameter used to accept simulations | Values accepted | |
|---|---|---|
| Number of HIV-HCV co-infections in 2014 | 450-850 | [1] |
| Annual number of new HIV-HCV co-infections (2014) | 100-150 | [2] |
| Incidence rate in 2012 through 2014 | 11-13 per 1,000 person-years | [3] |
| Incidence rate after DAA roll-out 2016 | 4 – 10 per 1000 person-years | [4, 5] |
| Reinfection rate in 2014 | 8 – 26.5% per year | [6, 7] |


1. Ard van Sighem LG, Colette Smit, Ineke Stolte, Peter Reiss. Monitoring Report 2014. Human Immunodeficiency Virus (HIV) Infection in the Netherlands In. Amsterdam: Stichting HIV Monitoring; 2014.
2. Hullegie SJ, van den Berk GE, Leyten EM, Arends JE, Lauw FN, van der Meer JT*, et al.* Acute hepatitis C in the Netherlands: characteristics of the epidemic in 2014. *Clin Microbiol Infect* 2016,**22**:209 e201-203.
3. Vanhommerig JW, Stolte IG, Lambers FA, Geskus RB, van de Laar TJ, Bruisten SM*, et al.* Stabilizing incidence of hepatitis C virus infection among men who have sex with men in Amsterdam. *J Acquir Immune Defic Syndr* 2014,**66**:e111-115.
4. Boerekamps A, Van den Berk GE, Fanny LN, Leyten EM, Van Kasteren ME, van Eeden A*, et al.* Declining HCV incidence in Dutch HIV positive men who have sex with men after unrestricted access to HCV therapy. *Clin Infect Dis* 2017.
5. Cotte L, Cua E, Reynes J, Raffi F, Rey D, Delobel P*, et al.* Hepatitis C virus incidence in HIV-infected and in preexposure prophylaxis (PrEP)-using men having sex with men. *Liver Int* 2018.
6. Lambers FAE, Prins M, Thomas X, Molenkamp R, Kwa D, Brinkman K*, et al.* Alarming incidence of hepatitis C virus re-infection after treatment of sexually acquired acute hepatitis C virus infection in HIV-infected MSM. *Aids* 2011,**25**:F21-F27.
7. Ingiliz P, Martin TC, Rodger A, Stellbrink HJ, Mauss S, Boesecke C*, et al.* HCV reinfection incidence and spontaneous clearance rates in HIV-positive men who have sex with men in Western Europe. *J Hepatol* 2017,**66**:282-287.
