# Supplementary material for: Early treatment of acute hepatitis C infection is cost-effective in HIV-infected men-who-have-sex-with-men
Source: PLoS One. 2019 Jan 10;14(1):e0210179. doi: 10.1371/journal.pone.0210179 (PMC6328146; doi:10.1371/journal.pone.0210179)
Supplement: S2 Table — (PDF) [file pone.0210179.s002.pdf]

Table S2: parameters used in the model

**Parameters of epidemic among HIV-positive MSM**	**Range [source/rationale]**
Annual new sexual partners	[Calibrated]
Highest risk group	20-100
Risk group 2	5-15
Risk group 3	1-4
Risk group 4	0.1 – 0.9
Proportion per risk group	[Calibrated]
Highest risk group	0.01 – 0.14
Risk group 2	0.02 - -0.2
Risk group 3	0-0.3
Risk group 4	0.4-0.9
Rate of assortative mixing	0-0.8 [Calibrated]
Patients in stage F3, F4 in 2002	10%-30% [Calibrated]
**Life Expectancy and mortality**	
Life expectancy HIV-infected men CD4>350	80 years [1]
Life expectancy HIV/HCV co- infected (F0-F3stage)	80 years
Life expectancy HIV/HCV co-infected compensated cirrhosis	0.024-0.055 per year [2]
Life expectancy HIV/HCV decompensated cirrhosis	0.019-0.35 per year [2]
**Disease progression**	
F0 to F1	0.098 – 0.122, per year [3]
F1 to F2	0.095 – 0.140, per year [3]
F2 to F3	0.097 – 0.159, per year [3]
F3 to Compensated cirrhosis	0.098 – 0.135, per year [3]
Compensated to decompensated cirrhosis	0.029 – 0.063, per year [2]
Compensated to decompensated cirrhosis	0.029 – 0.063, per year [2]
Cirrhosis to Hepatocellular carcinoma	0.01 – 0.03, per year [4]
Transplantations	0 per year
**Additional cost to delayed treatment stage**	

Parameters of epidemic among HIV-positive MSM	Range [source/rationale]
Cost of HCC (including hospitalization, treatment, surgery and care until death)	€67.591 – €233.573 per patient [5, 6]

1. May MT, Gompels M, Delpech V, Porter K, Orkin C, Kegg S*, et al.* Impact on life expectancy of HIV-1 positive individuals of CD4+ cell count and viral load response to antiretroviral therapy. *Aids* 2014,**28**:1193-1202.
2. Lopez-Dieguez M, Montes ML, Pascual-Pareja JF, Quereda C, Von Wichmann MA, Berenguer J*, et al.* The natural history of liver cirrhosis in HIV-hepatitis C virus-coinfected patients. *Aids* 2011,**25**:899-904.
3. Thein HH, Yi Q, Dore GJ, Krahn MD. Natural history of hepatitis C virus infection in HIV-infected individuals and the impact of HIV in the era of highly active antiretroviral therapy: a meta-analysis. *Aids* 2008,**22**:1979-1991.
4. Fattovich G, Stroffolini T, Zagni I, Donato F. Hepatocellular carcinoma in cirrhosis: incidence and risk factors. *Gastroenterology* 2004,**127**:S35-50.
5. Tapper EB, Catana AM, Sethi N, Mansuri D, Sethi S, Vong A*, et al.* Direct costs of care for hepatocellular carcinoma in patients with hepatitis C cirrhosis. *Cancer* 2016,**122**:852-858.
6. Baran RW, Samp JC, Walker DR, Smeeding JE, Young JW, Kleinman NL*, et al.* Costs and absence of HCV-infected employees by disease stage. *J Med Econ* 2015,**18**:691-703.
