# Supplementary material for: Early treatment of acute hepatitis C infection is cost-effective in HIV-infected men-who-have-sex-with-men
Source: PLoS One. 2019 Jan 10;14(1):e0210179. doi: 10.1371/journal.pone.0210179 (PMC6328146; doi:10.1371/journal.pone.0210179)
Supplement: S1 Fig — Comparison of the projected number of MSM that are diagnosed with HIV (black bullets and line) and the actual number of MSM diagnosed as reported by the Dutch HIV monitoring foundation. Comparison of the incidence rate of the Dutch population over time and the median simulations of our model. At T = 2014 the first DAAs were introduced in the Netherlands for F2/F3 patients and treatment in clinical trials. In 2015 DAAs became unrestricted available and in 2016 a new incidence data was available. (PDF) [file pone.0210179.s003.pdf]

S1 Calibration figures

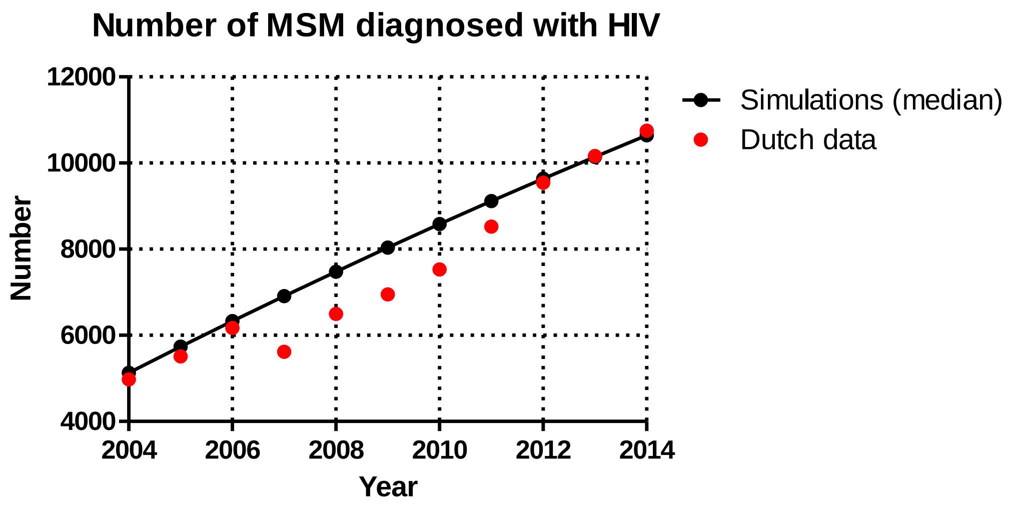

Comparison of the projected number of MSM that are diagnosed with HIV (black bullets and line) and the actual number of MSM diagnosed as reported by the Dutch HIV monitoring foundation

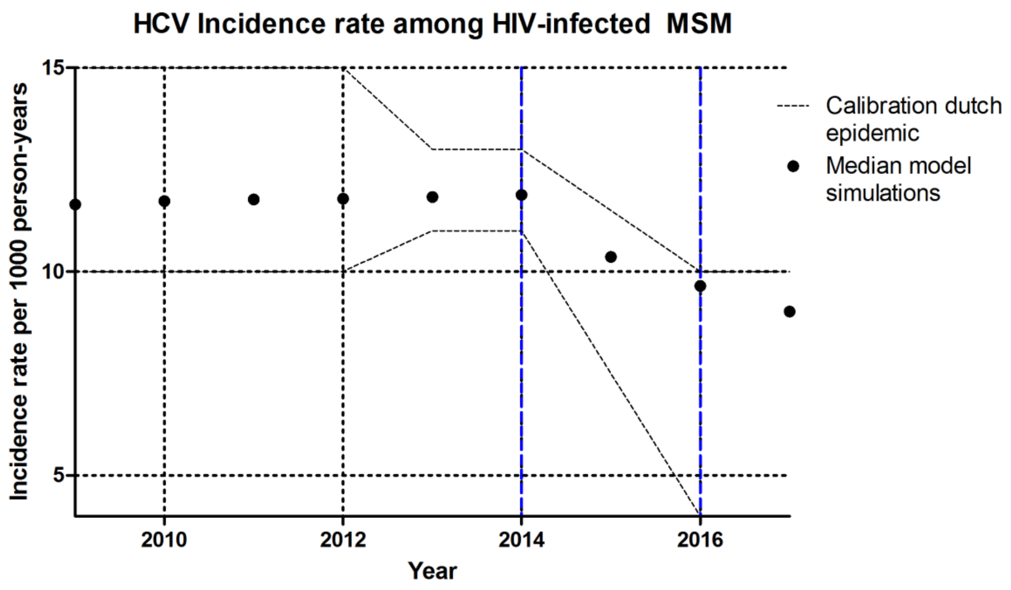

Comparison of the incidence rate of the Dutch population over time and the median simulations of our model. At T=2014 the first DAAs were introduced in the Netherlands for F2/F3 patients and treatment in clinical trials. In 2015 DAAs became unrestricted available and in 2016 a new incidence data was available[1].

1. Boerekamps A, van den Berk GE, Lauw FN, Leyten EM, van Kasteren ME, van Eeden A*, et al.* Declining Hepatitis C Virus (HCV) Incidence in Dutch Human Immunodeficiency Virus-Positive Men Who Have Sex With Men After Unrestricted Access to HCV Therapy. *Clin Infect Dis* 2018,**66**:1360-1365.
