# Supplementary material for: Early treatment of acute hepatitis C infection is cost-effective in HIV-infected men-who-have-sex-with-men
Source: PLoS One. 2019 Jan 10;14(1):e0210179. doi: 10.1371/journal.pone.0210179 (PMC6328146; doi:10.1371/journal.pone.0210179)
Supplement: S2 Fig — Individuals can be treated during an acute HCV infection in the immediate scenario, or treatment is delayed until possible spontaneous clearance, the so called chronic treatment scenario, or individuals are treated according to the delayed F2 treatment scenario. The stage of HCV infection. Individuals progress through the natural course of disease over time. Patients who do not spontaneously clear (Cl) their HCV infection can be put on DAA treatment. Abbreviations: DAA: direct-acting antivirals, F0-F3: fibrosis score METAVIR, HCC: hepatocellular carcinoma. (PDF) [file pone.0210179.s004.pdf]

S2. Simplified diagram capturing the HCV transmission model among HIV infected MSM evaluating different treatment scenarios.

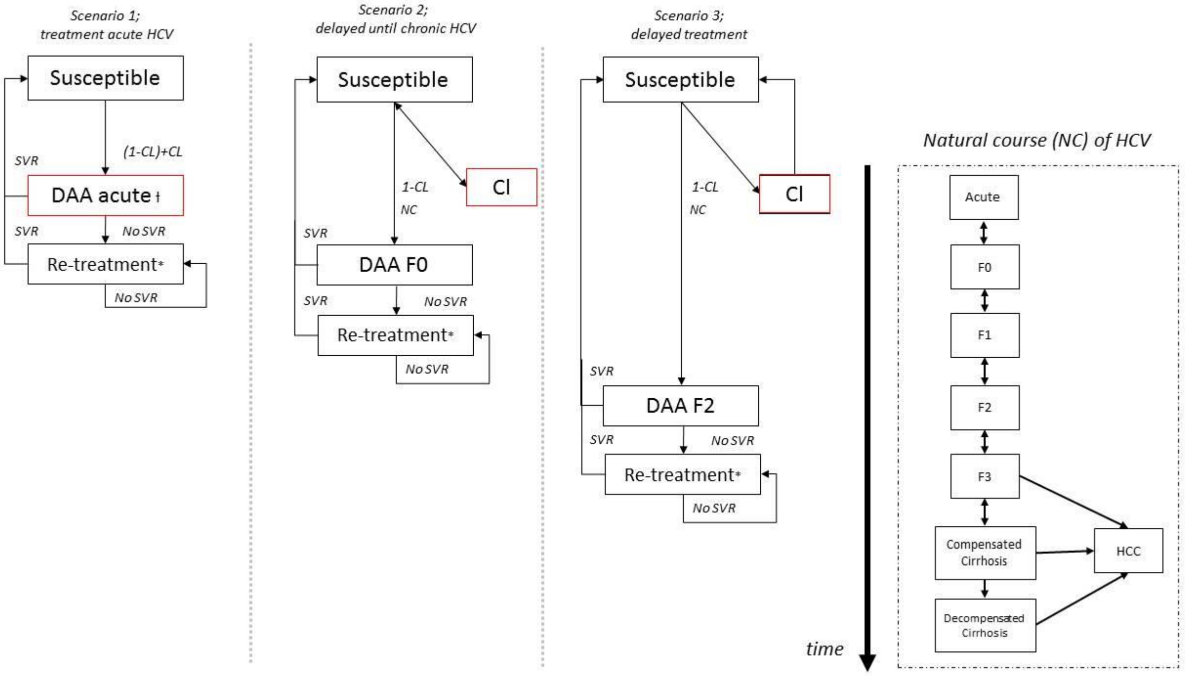

Individuals can be treated during an acute HCV infection in the immediate scenario, or treatment is delayed until possible spontaneous clearance, the so called chronic treatment scenario, or individuals are treated according to the delayed F2 treatment scenario. The stage of HCV infection. Individuals progress through the natural course of disease over time. Patients who do not spontaneously clear (Cl) their HCV infection can be put on DAA treatment. Abbreviations: DAA: direct-acting antivirals, F0-F3: fibrosis score METAVIR, HCC: hepatocellular carcinoma
