# Supplementary material for: Early treatment of acute hepatitis C infection is cost-effective in HIV-infected men-who-have-sex-with-men
Source: PLoS One. 2019 Jan 10;14(1):e0210179. doi: 10.1371/journal.pone.0210179 (PMC6328146; doi:10.1371/journal.pone.0210179)
Supplement: S3 Fig — Simulated Hepatitis C related hepatocellular carcinoma that can be avoided when treatment is administrated timely instead of delayed until F2 stage among HIV positive men-who-have-sex-with-men. F0 chronic, initiating treatment in the chronic phase of infection, waiting for the infection to spontaneously clear. T = 0 start of intervention. (PDF) [file pone.0210179.s005.pdf]

S3 Cumulative avoided hepatitis C related hepatocellular carcinoma compared to delayed F2 treatment

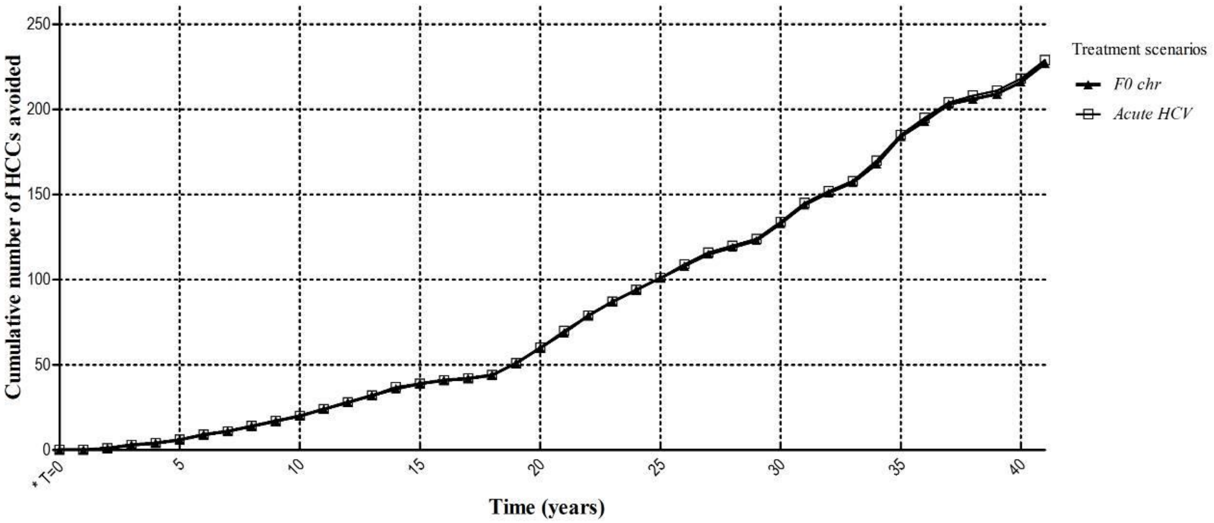

Simulated Hepatitis C related hepatocellular carcinoma that can be avoided when treatment is administrated timely instead of delayed until F2 stage among HIV positive men-who-have-sex-with-men. F0 chronic, initiating treatment in the chronic phase of infection, waiting for the infection to spontaneously clear. T=0 start of intervention.
