# Supplementary material for: Early treatment of acute hepatitis C infection is cost-effective in HIV-infected men-who-have-sex-with-men
Source: PLoS One. 2019 Jan 10;14(1):e0210179. doi: 10.1371/journal.pone.0210179 (PMC6328146; doi:10.1371/journal.pone.0210179)
Supplement: S4 Fig — In current guidelines pegylated-interferon is no longer recommended in the acute stage of hepatitis C virus (HCV) infection. However, individuals treated and cured with pegylated-interferon during the acute stage of HCV infection, could not further transmit HCV. The removal of pegylated-interferon and delaying DAA treatment until F2, therefore has a negative epidemiological impact. We conducted an uncertainty analysis, in which DAA treatment is delayed until F2 stage and pegylated-interferon is a possible option during the acute stage of HCV. In addition, the incidence decline of immediate and treatment during the F0 chronic stage is projected. Our analysis shows that the removal of pegylated-interferon during the acute stage of HCV infection and delaying treatment until F2 results in an increase of HCV incidence over time. Pegylated-interferon as an optional treatment in the acute stage while awaiting DAA treatment will stabilize the incidence. In contrast, early treatment with DAAs strongly reduces the HCV incidence over time. (PDF) [file pone.0210179.s006.pdf]

# S4 Influence of removal of pegylated-interferon as treatment of acute hepatitis C on the incidence among HIV-positive men-who-have-sex-with-men

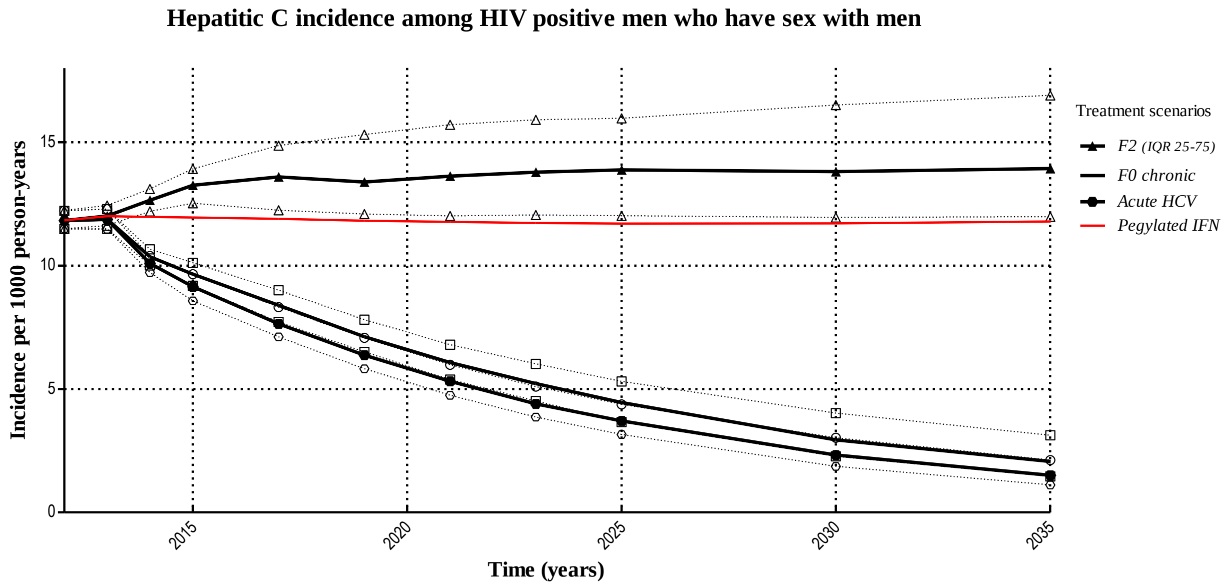


In current guidelines pegylated-interferon is no longer recommended in the acute stage of hepatitis C virus (HCV) infection. However, individuals treated and cured with pegylated-interferon during the acute stage of HCV infection, could not further transmit HCV. The removal of pegylated-interferon and delaying DAA treatment until F2, therefore has a negative epidemiological impact. We conducted an uncertainty analysis, in which DAA treatment is delayed until F2 stage and pegylated-interferon is a possible option during the acute stage of HCV. In addition, the incidence decline of immediate and treatment during the F0 chronic stage is projected. Our analysis shows that the removal of pegylated-interferon during the acute stage of HCV infection and delaying treatment until F2 results in an increase of HCV incidence over time. Pegylated-interferon as an optional treatment in the acute stage while awaiting DAA treatment will stabilize the incidence. In contrast, early treatment with DAAs strongly reduces the HCV incidence over time.
