# Supplementary material for: Early treatment of acute hepatitis C infection is cost-effective in HIV-infected men-who-have-sex-with-men
Source: PLoS One. 2019 Jan 10;14(1):e0210179. doi: 10.1371/journal.pone.0210179 (PMC6328146; doi:10.1371/journal.pone.0210179)
Supplement: S5 Fig — One-way sensitivity analysis of treatment immediately with DAAs after diagnosis versus delaying DAA treatment until F2 METAVIR stage with a cost-saving ICER of -8227/QALY. In both scenarios we simulate a group of individuals with undiagnosed/untreated HCV, the so called unidentified reservoir. We vary this from 0–100 and 0–2000. In addition, from 2016 onwards 6500 individuals are forced into the high risk groups of our model. (PDF) [file pone.0210179.s007.pdf]

S5 One-way sensitivity analysis of higher unidentified reservoir

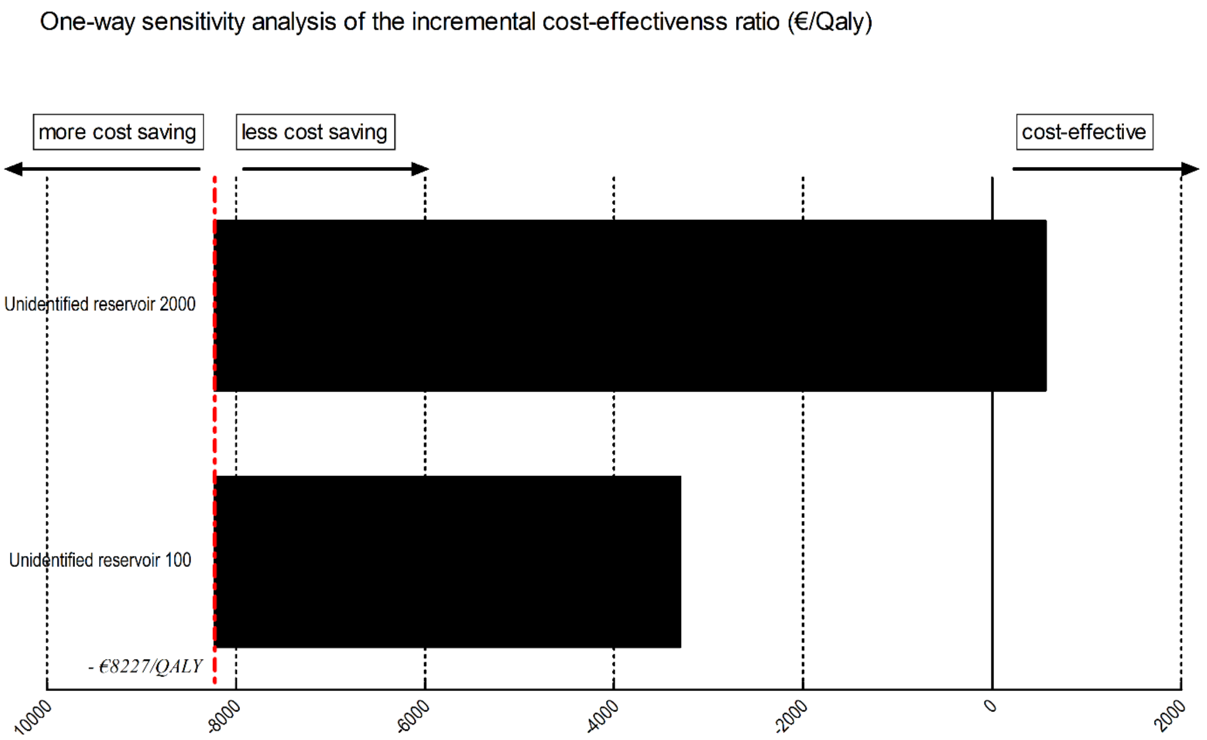

One-way sensitivity analysis of treatment immediately with DAAs after diagnosis versus delaying DAA treatment until F2 METAVIR stage with a cost-saving ICER of -8227/QALY. In both scenarios we simulate a group of individuals with undiagnosed/untreated HCV, the so called unidentified reservoir. We vary this from 0-100 and 0-2000. In addition, from 2016 onwards 6500 individuals are forced into the high risk groups of our model.
