# Supplementary material for: Early treatment of acute hepatitis C infection is cost-effective in HIV-infected men-who-have-sex-with-men
Source: PLoS One. 2019 Jan 10;14(1):e0210179. doi: 10.1371/journal.pone.0210179 (PMC6328146; doi:10.1371/journal.pone.0210179)
Supplement: S1 Text — (PDF) [file pone.0210179.s008.pdf]

# S1 Model description

The model is seeded in 2002 with 3,800 HIV-infected men-who-have-sex-men (MSM) of whom 3-10% were co-infected with hepatitis C (HCV). The state variables and the HCV transmission equations are shown below. The model includes four activity *i* based on the partner acquisition rate change: class 1 in which individuals have 20-100 HIV-infected partners per year, class 2 with 5-15 partners, class 3 with 1 -4 partners and class 4 with 0.1- 0.9 partners.

The model includes seven HCV infection stages: one stage including patients that are infected but that will clear HCV, five stages *j* of increasing severity of fibrosis (METAVIR stages F0, F1, F2, F3, F4). Stage F4 represents cirrhosis and is sub-divided into compensated cirrhosis (F4C) and decompensated cirrhosis (F4D). Stage F0 makes a distinction between patients that are diagnosed in a timely manner and who initiated treatment, patients that are not diagnosed, patients who are diagnosed but would have cleared treatment (necessary for scenario 1 and 2), patients who refuse treatment and patients in whom treatment is delayed (necessary for the third scenario). In the model until 2015, between 67% and 75% of patients with HIV that were acutely infected with HCV were treated for 24 weeks with pegylated interferon (PEG-IFN) and ribavirin (other patients declined treatment). Before 2012, chronically infected patients in METAVIR stages F2 through F4 were also treated with (PEG-IFN) and ribavirin. Between 2012 and 2015, boceprevir or telaprevir in addition to pegylated interferon and ribavirin, was prescribed to chronically infected patients.

After 2015, the model compares the epidemiological and economic impact of starting direct acting antivirals (DAA) immediately after HCV diagnosis, delayed until F0 chronic to await spontaneous clearance or delayed until METAVIR stage F2. When SVR is not reached the patient receives another 12 weeks of DAA treatment.
