# Supplementary material for: Early treatment of acute hepatitis C infection is cost-effective in HIV-infected men-who-have-sex-with-men
Source: PLoS One. 2019 Jan 10;14(1):e0210179. doi: 10.1371/journal.pone.0210179 (PMC6328146; doi:10.1371/journal.pone.0210179)
Supplement: S2 Text — (PDF) [file pone.0210179.s009.pdf]

S2 text. Technical model and equations

State variables

All individuals are MSM and infected with HIV

Variable	***Description***
S_i	Individuals not infected with HCV; sexual activity classes *i*=1..4
E_i	Entry rate HIV-infected MSM, sexual activity classes *i*=1..4
SPi	Individuals not infected with HCV that previously cleared HCV or were cured from HCV; sexual activity classes *i*=1..4
Cli,n	HCV infected individuals that are clearing HCV; sexual activity classes *i*=1..4<br>Different forms of clearing = n<br>1= clearing the virus and on treatment<br>2= clearing the virus and no treatment<br>3= clearing the virus and no diagnosis<br>4 = clearing the virus before diagnosis<br>B= after being infected
$F_{i,j,m}$	Stage of HCV infection; sexual activity classes *i*=1..4; stage of fibrosis *j*=0..4;<br>*m* is only defined for stage F0 (*j*=0) and stage F4 (*j*=4). At stage F0, *m*=1 are patients that will be treated during the acute stage and *m*=2 are patients that will not be treated. *m=3* are patients that are not diagnosed . At stage F4, *m*=1 is compensated cirrhosis and *m*=2 decompensated cirrhosis. *R*=reinfected
$Rx_{i,j,m,z}$	Patients receiving antiviral treatment for HCV; sexual activity classes *i*=1..4; stage of fibrosis *j*=0..4; consecutive number of DAA courses *z* used to treat a particular HCV infection z=1..3 (the value for z is set to zero after cure). At stage F4, *m*=1 is compensated cirrhosis and *m*=2 decompensated cirrhosis
$N_{i,j,z}$	Patients in whom treatment with DAA's did not result in a sustained virological response; sexual activity classes *i*=1..4; stage of fibrosis *j*=0..4; *z* is number of consecutive DAA courses that were unsuccessfully used to treat HCV z=2..3
$C_{i,m}$	Patients with cirrhosis that are not infected with HCV, sexual activity classes i=1..4, m=1 reflects compensated cirrhosis, m=2 decompensated cirrhosis
HCC	Hepatocellular carcinomas over time

Input variables

The values for the input variables are given in table 1 of the main text.

Variable	Description
λ_i	Force of infection or the rate by which individuals become infected with HCV in sexual activity group *i* (i=1..4)
μ	Mortality
μ_{comp}	Mortality, compensated cirrhosis
μ_{decomp}	Mortality, decompensated cirrhosis
$1/\tau$	Time to HCV clearance
SVRj	Sustained Virological Response (SVR) in fibrosis stage of infection *j* (j is 1..4)
SVR_{PEG}	Sustained Virological Response (SVR) for acutely infected patients that received treatment with pegylated interferon (only in the scenario's where DAAs are delayed until stage F2 or stage F3) during calibration
δ_j	duration of DAA treatment in stage of fibrosis *j*
δ_{PEG}	duration of pegylated interferon (only in the scenario's where DAAs are delayed until stage F2 or stage F3) during calibration
φ	proportion diagnosed with HCV
$\boldsymbol{\varepsilon}$	Proportion receiving treatment in acute stage of infection<br>E = 0 in delayed F2 scenario
Clear	Proportion clearing HCV
Θ_z	Time to start the zth course of DAA treatment; *z*=1, 2, 3
Δj,j+1	HCV progression rate by stage j=0..4
Δ_{decomp}	Progression rate from compensated to decompensated cirrhosis
χ	Percentage of patients that will develop an HCC over time
σ	Period to await spontaneous clearance to occur

Model equations

$\frac{dSi}{dt} = E_i - S_i * (\lambda_i + \mu)$

$\frac{dSPi}{dt} = \tau * (CL_{i,2} + CL_{i,2\ B} + CL_{i,13} + CL_{i,3B} + Clear) + \delta_j * (CL_{i,1\ B} + CL_{i,1)} + \sum_{j=0}^{j=3} \sum_{z=1}^{z=2} Rx_{\ i,j,z} * SVR_j * \delta_j + Rx_{i,3,3} * \delta_j * SVR_j - Sp_i * (\lambda_i + \mu)$

$\frac{dCli,n}{dt} = Clear * \varphi * \lambda_i * S_i * \boldsymbol{\varepsilon} - CL_{i,13} * \varphi * \boldsymbol{\varepsilon} - CL_{i,1} * (\tau + \mu)$

The following equations differ per scenario

1) immediate scenario

$\frac{dF\ i,0,1}{dt} = \varphi * \varepsilon * (1 - Clear) * \lambda_i * S_i + F_{i,0,3} * \varphi * \varepsilon - F_{i,0,1} * (\theta_z + \Delta_{0,1} + \mu)$

$\frac{dF\ i,0,1r}{dt} = \varphi * \varepsilon * (1 - \text{Clear}) * \lambda_i * SP_i - F_{0Reinf} * (\theta_z + \Delta_{0,1} + \mu)$

2) **the chronic treatment scenario**

$\frac{dF\ i,0,1}{dt} = \varphi * \varepsilon * (1 - \text{Clear}) * \lambda_i * S_i + F_{i,0,3} * \varphi * \varepsilon - F_{i,0,1} * \theta_z * \sigma + F_{i,0,1} (\Delta_{0,1} + \mu)$

$\frac{dF\ i,0,1r}{dt} = \varphi * \varepsilon * (1 - \text{Clear}) * \lambda_i * SP_i - F_{0Reinf} * \theta_z * \sigma + F_{0Reinf} * (\Delta_{0,1} + \mu)$

3) **the delayed F2 scenario**

$\frac{dF\ i,0,1}{dt} = \varphi * \varepsilon * (1 - \text{Clear}) * \lambda_i * S_i + F_{i,0,3} * \varphi * \varepsilon - F_{i,0,1} (\Delta_{0,1} + \mu)$

$\frac{dF\ i,0,1r}{dt} = \varphi * \varepsilon * (1 - \text{Clear}) * \lambda_i * SP_i - F_{0Reinf} * (\Delta_{0,1} + \mu)$

$\frac{dF\ i,1,}{dt} = (F_{i,0,1} + F_{i,0,2+} F_{i,0,1,r+} F_{i,0,3}) * \Delta_{0,1} - Fi,1 * (\Delta_{1,2} + \mu)$

$\frac{dRx\ i,j,z}{dt} = F_{i,j} * \theta_z + N_{i,j,z} * \rho - Rx_{i,j} * (\delta_j + \mu)$ for j ={2,3}, z={1,2}

$\frac{dN\ i,j,z}{dt} = Rx_{i,j,z-1} * \delta_j * (1 - SVR_j) + N_{i,j-1,z} * \Delta_{j-1,j} - N_{i,j,z} * (\Delta_{j,j+1} + \theta_z + \mu)$ for j={2,3}, z=2

Continuation of the model equations for all scenarios

*** For the delayed F2 scenario this formula is stated at the delayed F2 section**

$\frac{dF\ i,0,2}{dt} = (1 - \text{Clear}) * (1 - \varepsilon) * \varphi * \lambda_i * (S_i + SP_i) + F_{i,0,3} * \varphi * (1 - \varepsilon) - F_{i,0,2} * (\Delta_{0,1} + \mu)$

$\frac{dF\ i,0,3}{dt} = (1 - \text{Clear}) * (1 - \varphi) * \lambda_i * (S_i + SP_i) - F_{i,0,3} * \varphi - F_{i,0,3} * (\Delta_{0,1} + \mu)$

$\frac{dF\ i,1,}{dt} = (F_{i,0,1} + F_{i,0,2+} F_{i,0,1,r+} F_{i,0,3}) * \Delta_{0,1} - Fi,1 * (\theta z + \Delta_{1,2} + \mu)$ *

$\frac{dF\ i,j}{dt} = F_{i,j-1} * \Delta_{j-1,j} - F_{i,j} * (\theta_{z+} \Delta_{j,j+1} + \mu)$ for j={2,3}

$\frac{dF\ i,4,1}{dt} = F_{i,3} * \Delta_{3,4} - F_{i,4,1} * (\theta_z + \Delta_{decomp} + \mu_{comp})$

$\frac{dF\ i,4,2}{dt} = F_{i,4,1} * \Delta_{decomp} - F_{i,4,2} * (\theta_z + \mu_{decomp})$

$\frac{dRx\ i,j,z}{dt} = F_{i,j} * \theta_z + N_{i,j,z} * \rho - Rx_{i,j} * (\delta_j + \mu)$ for j ={0,1,2,3}, z={1,2} *

$\frac{dRx\ i,3,3}{dt} = N_{i,j,2} * \theta_z - Rx_{i,3,3} * (\delta_j + \mu)$

$$\frac{dRx\ i,4,1,z}{dt} = F_{i,4,1} * \theta_z + N_{i,4,1,z} * \theta_z – Rx_{i,4,1} * (\delta_j + \mu_{comp})$$

$$\frac{dRx\ i,4,2,z}{dt} = F_{i,4,2} * \theta_z + N_{i,4,2,z} * \theta_z – Rx_{i,4,2} * (\delta_j + \mu_{decomp})$$

$$\frac{dN\ i,j,z}{dt} = Rx_{i,j,z\text{-}1} * \delta_j * (1 – SVR_j) – N_{i,j,z} * (\Delta_{j,j+1} + \theta_z + \mu) \quad \text{for } j=0,\ z=2$$

$$\frac{dN\ i,j,z}{dt} = Rx_{i,j,z\text{-}1} * \delta_j * (1 – SVR_j) + N_{i,j\text{-}1,z} * \Delta_{j\text{-}1,j} – N_{i,j,z} * (\Delta_{j,j+1} + \theta_z + \mu) \quad \text{for } j=\{1,2,3\},\ z=2 \quad *$$

$$\frac{dN\ i,3,3}{dt} = Rx_{i,3,3} * \delta_j * (1 – SVR_j) + N_{i,3,3} * (\Delta_{3,4} + \theta_z + \mu)$$

$$\frac{dN\ i,4,1,z}{dt} = Rx_{i,4,1,z\text{-}1} * \delta_j * (1 – SVR_j) + N_{i,3,z} * \Delta_{3,4} – N_{i,4,1,z} * (\Delta_{decomp} + \theta_z + \mu_{comp}) \quad \text{for } z=\{2,3)$$

$$\frac{dN\ i,4,2,z}{dt} = Rx_{i,4,2,z\text{-}1} * \delta_j * (1 – SVR_j) + N_{i,4,1,z} * \Delta_{decomp} – N_{i,4,1,z} * (\theta_z + \mu_{decomp}) \quad \text{for } z=\{2,3\}$$

$$\frac{dCi,1}{dt} = \sum_{z=1}^{z=3} Rx_{i,4,1,z} * (\delta_j + SVR_j) – C_{i,1} * \mu_{comp}$$

$$\frac{dCi,2}{dt} = \sum_{z=1}^{z=3} Rx_{i,4,2,z} * (\delta_j + SVR_j) – C_{i,2} * \mu_{decomp}$$

$$\frac{dCli,1B}{dt} = Clear * \varphi * \varepsilon * \lambda_i * Spi + Cl_{i,3B} * \varphi * \varepsilon – Cl_{i,1\ B\ *}\ (\tau + \mu)$$

$$\frac{dCli,2}{dt} = Clear * \varphi * (1\text{-}\varepsilon) * \lambda_i * Si + Cl_{i,3} * \varphi * (1\text{-}\ \varepsilon) – Cl_{i,2} * (\tau + \mu)$$

$$\frac{dCli,2B}{dt} = Clear * \varphi * (1\text{-}\varepsilon) * \lambda_i * SPi + Cl_{i,3B} * \varphi * (1\text{-}\ \varepsilon) – Cl_{i,2B} * (\tau + \mu)$$

$$\frac{dCli,3}{dt} = Clear * \varphi * \varepsilon * (1\text{-}\ \lambda_i) * Si + Cl_{i,3} * \varphi\ – Cl_{i,3} * (\tau + \mu)$$

$$\frac{dCli,3B}{dt} = Clear * \varphi * \varepsilon * (1\text{-}\ \lambda_i) * SPi + Cl_{i,3B} * \varphi\ – Cl_{i,3B} * (\tau + \mu)$$

$$\frac{dCli,4}{dt} = Clear * \lambda_i * (Si + SPi) – Cl_{i,4} * (\tau + \mu)$$

$$\frac{dHCC}{dt} = \chi * \sum_{j=3}^{j=4} \sum_{m=1}^{m=2} F i,j,m + \sum_{j=3}^{j=4} \sum_{m=1}^{m=2} \sum_{z=1}^{z=3} Rx i,j,m,z + \sum_{j=3}^{j=4} \sum_{m=1}^{m=2} \sum_{z=1}^{z=3} N i,j,m,z$$

Force of infection

The equation for the force of infection includes a mixing matrix $M_{i,j}$ for infected individuals. The elements of the matrix are i,j and represent the probability that an individual with *i* new partnerships per year will form a new partnership with a member who has *j* new partners. The rate at which the sexual partner changes for individuals in each sexual activity group *i* is expressed as C_i. N_i = number of individuals in sexual activity class *i*. The values of the mixing matrix depend on the degree of mixing ε. This degree can be fully assortative (ε=1), where partnerships are only formed within the same activity class. Or fully random (ε=0) where partnerships are formed between different activity classes [15, 16].

$$M_{i,j} = \varepsilon\delta + \frac{(1-\varepsilon)CjNj}{\sum_{i=1}^{4} CiNi}$$

Where δ is Kronecker's delta, with $\delta = 1$ when $i = j$, and δ=0 when $i \neq j$.

The force of infection (λ) is calculated using the following formula:

$$\lambda_i = \lambda_{i1} * (Cl_{i + j,0,m} + {}_{j,m} + {}_{j,4,m} + {}_{j})$$

In which λ_i is the force of infection due to contact with an infected person. Similarly, λ_{i1} is the force of infection due to contact with one person that is infected with HCV and not on treatment.

1. Boerekamps A, van den Berk GE, Lauw FN, Leyten EM, van Kasteren ME, van Eeden A*, et al.* Declining Hepatitis C Virus (HCV) Incidence in Dutch Human Immunodeficiency Virus-Positive Men Who Have Sex With Men After Unrestricted Access to HCV Therapy. *Clin Infect Dis* 2018,**66**:1360-1365.
2. Ard van Sighem LG, Colette Smit, Ineke Stolte, Peter Reiss. Monitoring Report 2014. Human Immunodeficiency Virus (HIV) Infection in the Netherlands In. Amsterdam: Stichting HIV Monitoring; 2014.
3. Hullegie SJ, van den Berk GE, Leyten EM, Arends JE, Lauw FN, van der Meer JT*, et al.* Acute hepatitis C in the Netherlands: characteristics of the epidemic in 2014. *Clin Microbiol Infect* 2016,**22**:209 e201-203.
4. Vanhommerig JW, Stolte IG, Lambers FA, Geskus RB, van de Laar TJ, Bruisten SM*, et al.* Stabilizing incidence of hepatitis C virus infection among men who have sex with men in Amsterdam. *J Acquir Immune Defic Syndr* 2014,**66**:e111-115.
5. Boerekamps A, Van den Berk GE, Fanny LN, Leyten EM, Van Kasteren ME, van Eeden A*, et al.* Declining HCV incidence in Dutch HIV positive men who have sex with men after unrestricted access to HCV therapy. *Clin Infect Dis* 2017.
6. Cotte L, Cua E, Reynes J, Raffi F, Rey D, Delobel P*, et al.* Hepatitis C virus incidence in HIV-infected and in preexposure prophylaxis (PrEP)-using men having sex with men. *Liver Int* 2018.
7. Lambers FAE, Prins M, Thomas X, Molenkamp R, Kwa D, Brinkman K*, et al.* Alarming incidence of hepatitis C virus re-infection after treatment of sexually acquired acute hepatitis C virus infection in HIV-infected MSM. *Aids* 2011,**25**:F21-F27.
8. Ingiliz P, Martin TC, Rodger A, Stellbrink HJ, Mauss S, Boesecke C*, et al.* HCV reinfection incidence and spontaneous clearance rates in HIV-positive men who have sex with men in Western Europe. *J Hepatol* 2017,**66**:282-287.
9. May MT, Gompels M, Delpech V, Porter K, Orkin C, Kegg S*, et al.* Impact on life expectancy of HIV-1 positive individuals of CD4+ cell count and viral load response to antiretroviral therapy. *Aids* 2014,**28**:1193-1202.
10. Lopez-Dieguez M, Montes ML, Pascual-Pareja JF, Quereda C, Von Wichmann MA, Berenguer J*, et al.* The natural history of liver cirrhosis in HIV-hepatitis C virus-coinfected patients. *Aids* 2011,**25**:899-904.
11. Thein HH, Yi Q, Dore GJ, Krahn MD. Natural history of hepatitis C virus infection in HIV-infected individuals and the impact of HIV in the era of highly active antiretroviral therapy: a meta-analysis. *Aids* 2008,**22**:1979-1991.
12. Fattovich G, Stroffolini T, Zagni I, Donato F. Hepatocellular carcinoma in cirrhosis: incidence and risk factors. *Gastroenterology* 2004,**127**:S35-50.
13. Tapper EB, Catana AM, Sethi N, Mansuri D, Sethi S, Vong A*, et al.* Direct costs of care for hepatocellular carcinoma in patients with hepatitis C cirrhosis. *Cancer* 2016,**122**:852-858.
14. Baran RW, Samp JC, Walker DR, Smeeding JE, Young JW, Kleinman NL*, et al.* Costs and absence of HCV-infected employees by disease stage. *J Med Econ* 2015,**18**:691-703.
15. Garnett GP, Anderson RM. Factors controlling the spread of HIV in heterosexual communities in developing countries: patterns of mixing between different age and sexual activity classes. *Philos Trans R Soc Lond B Biol Sci* 1993,**342**:137-159.
16. Baggaley RF, Garnett GP, Ferguson NM. Modelling the impact of antiretroviral use in resource-poor settings. *PLoS Med* 2006,**3**:e124.
